# Technological Application of Tannin-Based Extracts

**DOI:** 10.3390/molecules25030614

**Published:** 2020-01-30

**Authors:** Maria Fraga-Corral, Paula García-Oliveira, Antia G. Pereira, Catarina Lourenço-Lopes, Cecilia Jimenez-Lopez, Miguel Angel Prieto, Jesus Simal-Gandara

**Affiliations:** 1 Nutrition and Bromatology Group, Analytical and Food Chemistry Department, Faculty of Food Science and Technology, University of Vigo, Ourense Campus, E-32004 Ourense, Spain; maria.fraga.corral@hotmail.es (M.F.-C.); paula.garcia.oliveira@uvigo.es (P.G.-O.); anthea.rande@gmail.com (A.G.P.); c.lopes@uvigo.es (C.L.-L.); cecilia.jimenez.lopez@uvigo.es (C.J.-L.); mprieto@uvigo.es (M.A.P.); 2 Centro de Investigação de Montanha (CIMO), Instituto Politécnico de Bragança, Campus de Santa Apolonia, 5300-253 Bragança, Portugal

**Keywords:** tannins, natural molecules, additives, traditional utilization, industrial application, human and animal health

## Abstract

Tannins are polyphenolic compounds naturally found in vegetables. Their presence in nature has prompted their historical use in many different ways. The revision of their traditional utilization has allowed their further modification aiming for an industrial application. Sometimes these modifications have implied the addition of harmful substances such as formaldehyde, classified as a carcinogen of category B1. In other cases, these natural tannins have been replaced by synthetic compounds that threaten human and animal health and damage the environment. Therefore, currently, both academy and industry are searching for the substitution of these unsafe complexes by the increasing inclusion of tannins, natural molecules that can be obtained from several and diverse renewable resources, modified using harmless additives. To achieve promising results, cost-efficient and eco-friendly extraction methods have been designed. Once these green alternatives have been isolated, they have been successfully applied to many fields with very assorted aims of utilization such as coagulants, adhesives, floatation agents, tannings, dyes, additives, or biomolecules. Therefore, this review offers a global vision of the full process that involves the tannin’s technological application including an overview of the most relevant tannin sources, effective extraction methods, and their utilization in very diverse fields.

## 1. Introduction

Polyphenols comprise a vast family of secondary metabolites which are stored in vacuoles of vegetal cells such as esters or glycosides. Even though this family of compounds is huge, they share some common properties, such as the formation of coloured complexes with iron salts, oxidation by potassium permanganate in alkaline media, and easy electrophilic aromatic substitution-coupling with diazonium salts and aldehydes. Tannins are considered polyphenols with high molecular weights. Thus, they possess typical features from the polyphenols group, but not only that. The presence of multiple functional groups in the chemical structure of tannins, such as hydroxyls, provides them with the ability to create bonds to reach a stable cross-linked association within different molecules, such as proteins or carbohydrates. This unique characteristic lets them be differentiated from the common group of polyphenols [1,2]. 

**Figure 1:**
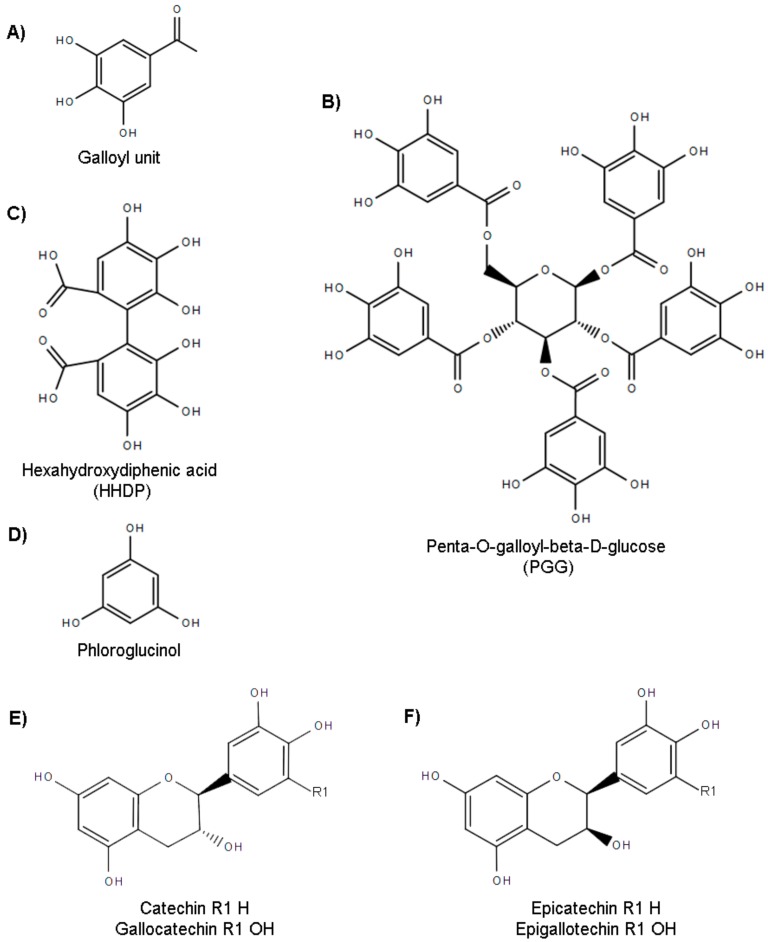
Chemical structure of common molecules found along with the tannins class. (**A**) Galloyl unit, (**B**) penta-*O*-galloyl-beta-d-glucose (PGG), (**C**) hexahydroxydiphenic acid, (**D**) phloroglucinol, (**E**) catechin and gallocatechin, (**F**) epimers of catechin and gallocatechin.

Historically, tannins have been divided into two main groups of polyphenols, hydrolysable and condensed ones, this last one is also known as proanthocyanidins. Both groups were usually described to be synthesized by higher plants, mostly. However, over the last decades the structural classification, based on their chemical structure, and the description of producers has been updated. Nowadays, algae, specifically those belonging to the Phaeophyceae class, have been included as organisms capable of synthesizing an exclusive class of tannins, phlorotannins which the common structural base is the phloroglucinol (Figure 1D). Regarding their chemical classification, they can be currently divided into five main categories: Gallotannins, ellagitannins, complex tannins, condensed tannins, and phlorotannins [3,4,5,6]. 

Galloyl units (Figure 1A) are the scaffold over which the first two types of tannins are built but they are differentiated by the chemical unions that provide them their final structure. Gallotannins (Figure 1E,F) are polymers of galloyl units that bond to polyol-, catechin-, or triterpenoid units. Pentagalloylglucose, (PGG, Figure 1B) represents a simple example of this group, it consists of five galloyl units that bond by five respective esters to a molecule of glucose. More complex molecules involve the formation of galloyl chains that can enlarge its size. These chains are built by coupling galloyl units through meta- or para-depside bonds. This type of linkage is generally described as the union of two esterified monocyclic aromatic units, galloyl in this case which is usually bound through hydroxyl groups [5,6,7,8]. 

Galloyl units are also the main components of ellagitannins but, in this case, the oxidation of at least two galloyl moieties leads to their coupling by carbon–carbon bonds, and results in the formation of hexahydroxydiphenic acid (HHDP, Figure 1C). The simplest molecule belonging to ellagitannins is the ellagic acid that happens after HHDP is spontaneously lactonized. More complex ellagitannins are obtained by esterification of HHDP units which increase their complexity by coupling themselves to different hydrolysable tannins by intermolecular oxidation reactions [5,6,7]. 

Complex tannins are formed by one unit of these previous categories; it means a gallotannin or an ellagitannin, which is assembled through a glycosidic bond to a flavan-3-ol (catechin monomer). Catechins are usually linked at a carbon position of an open glucose molecule. Acutissimin A and eugenigrandin A are two examples of complex tannins in which a gallocatechin or catechin, respectively, are complexed through a carbon–carbon bond to an ellagitannin unit [5,9].

Condensed tannins are oligomers or polymers of flavan-3-ols coupled by carbon–carbon bonds, that are usually formed by the linkage of C–4 from a catechin to a C–8 of the next one, even though C–4 to C–6 bonds are also possible they are not so common [5]. Tannins polymerization occurs by condensation of catechins, when the number of condensed units is between two and ten, they are considered as flavolans. Additional reactions, such as hydroxylation, methylation, glycosylation, or galloylation increase the complexity of the initial molecule. Based on the chemical structure and the reactions that occurred in the compound, they allow dividing them into subgroups. For example, procyanidins comprise molecules with hydroxyl groups at 3′, 3′, 4′, 5′, and 7′ positions and are differentiated from prodelphinidins since the latter possesses an additional hydroxyl at 5’ position [5,6,10,11].

The basic unit of the phlorotannins is 1,3,5-trihydroxybenzene, also known as phloroglucinol (Figure 1D). Phloroglucinol monomers can be incorporated into the molecule using linear and/or branch linkages to form oligomers or polymers that may achieve molecular masses up to 650 kDa. Phlorotannins can be classified based on the structural bonds found in their molecules and there are three main groups: Fucols (C–C bonds), phloroethols (C–O–C bonds), and fucophloroethols (combine C–C with C–O–C bonds). Different phlorotannins have been isolated. Some examples are presented showing the increasing number of phloroglucinol units (PUs), directly related with their increasing structural complexity: Eckol or dioxinodehydroeckol (3PUs); fucodiphloroethol G, 7-phloroeckol, tetrafucol A, tetraphlorethol B or fucodiphlorethol (4 PUs), phlorofucofuroeckol A, and B (5 PUs); dieckol, 6,6′-bieckol, fucotetraphlorethol A, hexafucol B, and fucotetraphlorethol D (6 PUs); and heptaphlorethol and heptafucol (7 PUs) [12,13]. 

The diversity of structures that can be found along this family of polyphenols justifies the wide molecular weight range of tannins, from 500 up to 20,000 Daltons. The same diversity together with their chemical properties confer them different uses, such as coagulants, adhesives, flotation and dye agents, food or drinking additives, and they can be also applied to medical and veterinary fields. Additionally, the high content of tannins in plants—they can represent up to 20% of dry weight, and they are the fourth secondary metabolite synthesized by higher plants, after cellulose, hemicelluloses, and lignin—explain their use since it represents a valuable material from an economic and natural source. Therefore, this review aims to provide detailed information about the organisms with major rates of tannins production, the best practices to efficiently extract them and their multiple industrial and technological applications. 

## 2. Major Sources of Tannins for Technological Applications

The variety of plants in which tannins have been found is huge. They possess important ecological roles since they participate in different processes such as litter decomposition, metal complexation, or microbial activity. In plants, their synthesis is usually associated with defensive responses against herbivores (due to the astringent taste and characteristic smell), pathogens or UV-A and UV-B radiation. Additionally, as explained before, tannins are polyphenols that are stored in vacuoles, thus they have been found nearly in all vegetal tissues: Bark, wood, leaves, fruit, roots, seeds, or galls [5,6,14,15]. While hydrolysable tannins are present in few dicotyledons species, the natural presence of condensed ones is much more abundant, thus they represent the major and more valuable source of commercial tannins [5]. Hydrolysable tannins, such as ellagitannins, are found in combination with condensed ones in different proportions in nuts. In the case of *Juglans regia,* the concentration of hydrolysable and condensed tannins is quite low (0.05 and 0.06 mg/g, respectively). In *Carya illinoinensis,* hydrolysable tannins are found in low amounts (0.22 mg/g) while condensed ones present wide ranges of concentration (0.4–876 mg/g), mostly depending on target tissue [2,16,17]. These tannins are also present in concentrations ranging from 0.03 to 3.26 mg/g in fruits, for example in *Ribes nigrum*, *Fragaria × ananassa* Duchesne, *Punica granatum, Psidium* spp., *Rubus fruticosus,* and *R. occidentalis* (Table 1). 

The presence of proanthocyanidins in these fruits represents less than 20% of total tannins, while the genus *Rubus* is the most productive in terms of ellagitannins. Gallotannins are less frequent but they have been detected at concentrations in the range of 1.0 mg/g in mango (*Mangifera indica*) and 0.3 mg/g in almonds (*Prunus dulcis*), in the latter one mixed with other tannins [2,16,18]. Usually, this is the most common composition in nature, a mixture of tannins in which the proportion of condensed tannins is much higher than hydrolysable ones. This is the case of many legumes and tree species. The legume content of condensed tannins is very variable depending on the tissue and its stage (for example, immature or mature) and the technique employed for its analysis. This variability was displayed in the wide quantification ranges of concentrations of the total content of condensed tannins that were obtained from *Desmodium ovalifolium, Gliricidia sepium, Manihot esculenta,* and *Arachis pintoi*: 57–273, 25–186, 26–169, and 40–186 mg/g of dry weight, respectively (Table 1) [19,20].

The same variability in data occurs with tree species, mostly due to the application of different analytical and quantitative methods, diversity of analyzed tissues, geographical origin of samples, and recollecting period. In the case of *Castanea sativa*, it has been found to contain levels of about 0.1–0.2 µg/g of edible portions of the chestnut fruit. However, when other tissues (leaves, wood, or bark) have been analysed much higher amounts of tannins, besides other phenols, were detected, achieving total concentrations up to 167 mg/g in optimal conditions at the bark (Table 1). Ellagic acid was present at different levels, ranging from 0.7 to 89 mg/g, depending on the analysed tissue, being the most representative wood, bark, and fruit [2,21,22]. 

The presence of tannins is highly variable among different species of the same genus. That is the example of *Terminalia horrida*, *T. chebula*, and *T. bellerica*. A study identified 34 polyphenols, mainly hydrolyzable tannins, belonging to five classes (gallic acid and simple gallates; chebulic ellagitannins; non-chebulic ellagitannins; ellagic acid and derivatives; and ellagic glycosides) and quantified their content in fruits obtained from *T. horrida*, *T. chebula,* and *T. bellerica* in 822, 126, and 224 mg/g (Table 1) [23]. Quercus is another genus in which different species and different locations imply important changes in the composition of their tissues. Samples obtained from specimens of *Quercus robur, Q. petraea,* and *Q. alba* collected from Eastern Europe (Ukraine, Moldavia, and Romania), France and the United States of America (USA, Kentucky, Indiana, Missouri, and Pennsylvania) were analyzed. The determination of total ellagitannins in wood provided significant differences between species: For *Q. robur* 47.26 mg/g, for *Q. petraea* 30.88 mg/g, and for *Q. alba* 8.36 mg/g. Differences between locations are likely to be due to the sample composition since it provides values of total ellagitannins of 31.20 mg/g for Eastern Europe (64 samples from *Q. robur* and 38 from *Q. petraea*), 42.39 mg/g for France (158 samples from *Q. robur* and 118 from *Q. petraea*), and 8.36 mg/g for USA (56 samples from *Q. alba*) which are very concordant with amounts obtained for each species [24]. However, another work confirmed this inter-continental difference observed. It evaluated the variation on the detection of ellagitannins using wood from diverse species of *Quercus* and different locations and applying or not toasting. The process of toasting displayed diminution on the content of ellagitannins. Results obtained from non-toasted tissues are consistent with the work previously mentioned since total tannins (the sum of castalagin, vescalagin, roburin A–E, and grandinin) detected in *Q. alba* from USA (Missouri) was 7.99 mg/g, and *Q. robur* and *Q. petraea* from Europe was about 23.5 ± 1.7 mg/g (Table 1) [25].

On occasions, studies focused their attention on just one class of compounds, therefore, compiling information about the total composition of one species became difficult. *Betula* spp. leaves were analyzed in one work that determined their content in soluble and bound condensed tannins in concentrations up to 103 and 40 mg/g, respectively. Another study, however, points to the optimization of the extraction of hydrolysable tannins depending on the chosen solvent, storage conditions, and dry technique obtaining ranges from 0.72 to 5.92 mg/g for insoluble ellagitannins and 11.58 up to 60.67 mg/g for the total composition of hydrolysable tannins (Table 1) [33]. Instead, few species contain a similar amount of tannins even though they are taxonomically distant and extraction or detection protocols used by authors were different. This is the case of *Eucalyptus globulus, Pinus sylvestris,* and *Acacia* sp., all of them possess a similar range of phenolic compounds (Table 1). *Eucalyptus globulus* contains about 132 mg/g of total phenols corresponding more than 70% of tannins (gallotannins, ellagitannins, and phloroglucinols) [35]. In *Pinus sylvestris,* the phenolic content is about 85–100 mg/g from which 80% are condensed tannins [34]. When six diverse species of Acacia (*A. angustissima, A. drepanolobium, A. nilotica, A. polyacantha, A. tortilis,* and *A. senegal*) collected from north-western Tanzania were examined, obtained concentrations of total extractable tannins ranged from 84 to 256 mg/g. The proportion of condensed tannins included in that total amount was highly variable from 20% to 100%. The highest content on condensed tannins was found in *A. drepanolobium* and *A. polyacantha* with values of 84.2 and 98.3 mg/g [34,35,36]. In the same range was the quantification of another species of Acacia known as mimosa (*A. mearnsii*) with a concentration of 235.4 mg/g of total condensed tannins when determined using the colorimetric assay butanol-HCl. However, when the HPLC–MS detection was applied, this value was 108 mg/g of procyanidin C1 equivalents. Inversely, the analysis of condensed tannins present in *Caesalpinia spinosa* using the colorimetric assay showed a low concentration of condensed tannins, 4.6 mg/g, but results obtained with HPLC–MS displayed a high concentration of hydrolysable tannins, 647 mg/g of epigallocatechin gallate equivalents. In the case of quebracho samples, *Schinopsis lorentzii*, similar results were analyzed using the butanol-HCl assay values of 122.7 mg/g of condensed tannins that were achieved while HPLC–MS results displayed 164.3 mg/g of procyanidin C1 equivalents (Table 1) [37].

In general terms, tree species such as *Acacia* sp. or *Terminalia* sp. are important sources of both hydrolysable and condensed tannins. The most relevant species for hydrolysable tannins have been demonstrated to be *Caesalpinia spinosa* while nutshells from *Carya illinoinensis* seem to represent a potential source of condensed tannins. However, most of the literature results are based on colorimetric and spectrophotometric techniques that sometimes overestimate the content of tannins; therefore, data based on analytical methods should be considered when selecting a vegetal matrix as a source of tannins.

## 3. Efficient Extraction Procedures for Technological Application of Tannins: Rapid and Economic 

### 3.1. Solid/Liquid Extraction

Traditionally, the simplest method for recovering tannins is the solid/liquid extraction, where a liquid solvent passes through a pulverized tissue and dissolves soluble compounds of the matrix without applying any other assisting mechanism. Some examples of this system are the maceration, decoction, and Soxhlet extraction. Different solvents have been tested over the years. As a first extraction step, dichloromethane or hexane may be used to remove chlorophylls and lipids. After that, the second step is to selectively extract tannins. The solubility of tannins is variable, so depending on the target compound, solvents with different relative polarities may be chosen, such as water, ethanol, acetone, or methanol [8]. Condensed tannins have limited solubility in a polar organic solvent, whereas water or ethanol are commonly used solvents for the extraction of hydrolysable tannins [8,35]. However, the use of solvents such as acetone or hexane implies the generation of residues, which represents an environmental threat that can directly affect both human and animal health threats. Therefore, water, that allows extracting concentrations of tannins ranging from 29 to 887 mg/g of dried extract, is the preferred solvent at an industrial scale [38]. This aqueous-based procedure is the simplest to perform, and residues generated do not affect so negatively on the environment. In addition, the recovery of tannins is quite elevated, revealing this extraction as an efficient technique, in both economic and environmental terms. The main disadvantage of this method is the high amount of solvent required [39].

Nowadays, solid/liquid extractions use water, usually in combination with other reagents such as ethanol, sodium hydroxide (NaOH), sodium carbonate (Na_2_CO_3_), sodium bisulphite (NaHSO_3_), or sodium sulphite (Na_2_SO_3_) (Table 2) [40,41,42]. The reduction of pH using alkalis and the incorporation of ethanol has been described to improve the extraction efficiency. Another factor enhancing tannins extraction is the application of high temperatures. Nevertheless, when tannins are described to be thermally unstable, the extraction duration which generally lasts 2–6 h at high temperatures may be extended until 24–48 h accompanied by a reduction of the temperature [38,39]. Once the process has been finished, the solid fraction is often separated from the extract by a filtration system and then lyophilized [40,41,42]. 

A further step on solid/liquid extraction is the substitution of conventional solvents by ionic liquids, a novel technology still in development. Ionic liquids are organic salts in liquid state, they present insignificant vapour pressure, elevate thermal stability, and the ability to dissolve a huge number of substances. Ionic liquids have been also applied in combination with ultrasounds. The recovery of tannins using ionic liquids ranges from 3.4 up to 630 mg/g of dried extract (Table 2) [38,43]. When compared with water, ionic liquids have been demonstrated to achieve similar results but significantly higher, as in the case of *Acacia catechu* [44]. Extraction times are shorter than 4.5 h since when longer protocols are applied the concentration of tannins is reduced due to its decomposition. In this kind of extraction, temperatures are normally under the boiling point of the solvent; in fact, it has been developed even at room temperature [38,44]. 

These ionic liquid-based methods represent an easy and rapid tool for an efficient extraction of tannins since it may reduce the experimental time of extraction compared with those using conventional solvents. However, these solvents are expensive for application on the industrial scale [38]. 

### 3.2. Supercritical Extraction

Supercritical fluids are substances submitted to the highest values of temperature and pressure—known as critical point—at which they can exist in a gas–liquid equilibrium. In this situation, the substance presents properties for both physical states [38,45]. The most used solvent in supercritical fluid extraction is carbon dioxide (CO_2_), due to its non-toxicity, availability, and low critical temperature and pressure, among other advantages [39,45]. However, CO_2_ is a non-polar molecule while tannins are considered polar compounds. Thus, to enhance the solubility of tannins and the efficiency of their extraction, polar co-solvents are used, such as ethanol, methanol, and aqueous mixtures [45,46,47]. Different factors are involved in the throughput of supercritical extraction such as the nature of the solvent and co-solvent, flow rate, extraction time, moisture content of raw material, particle size, and especially pressure and temperature. Temperature and pressure are the most cited parameters considered for the optimization of the process, independently of the target molecule or selected matrix or solvent [48]. The range of values for these parameters that yielded the best extraction efficiencies for tannins varied between 40 and 80 °C for temperature and from 10 to 65.5 MPa for pressure (Table 2) [38]. Regarding pressure, higher values have been reported to enhance the amount of tannins extracted, as the solubility of compounds increases. In the case of temperature, lower values are preferred to avoid thermal degradation [38,47]. To assure good levels of extraction, compounds solubility has to be enhanced (through adding co-solvents of manipulating pressure values) and the matrix impregnation [47]. The extraction levels range from 26.38 to 770 mg/g of dried extract (Table 2) [45,46]. 

The supercritical extraction method shows several advantages such as the use of mild temperatures, the short times of extraction and low toxicity levels, in terms of generated residues [45]. On the other hand, the main disadvantage is the high investment necessary to acquire the equipment [38].

### 3.3. Pressurized Water Extraction

Pressurized water extraction has been also applied to extract tannins. It is based on the use of water at high temperature and pressure, but under the critical point (achieved at limiting conditions: 374 °C, 22.1 MPa) [38,49]. Usually, to perform the extraction, water is maintained at conditions under the critical point, which means in the liquid state [39,49]. Tannins extraction rates with this method are very wide ranging from 52.9 to 381.9 mg/g of dried extract (Table 2) [50,51]. The main advantage of this technique is the reduction of the extraction time to 5–60 min [38] which entail a reduction of the potential degradation of tannins, since it has been directly related to long extraction times [39].

The saving time and the use of water, a non-toxic substance, are the main advantages of this method [38,49]. Moreover, this method allows modulating temperature, pressure, or the co-solvent, to permit extracting different classes of tannins, selectively. However, as in the case of the use of supercritical fluids, it is not a cost-effective technique since it depends on expensive equipment [39].

### 3.4. Microwave-Assisted Extraction

This method combines the use of traditional solvents and heating by microwaves [39]. Intracellular content is heated and evaporated, producing strong pressure. This pressure breaks the cell wall and membranes, liberating intracellular content into the solvent [59,61]. Water, methanol, and ethanol are the solvents recommended for this extraction [39]. The frequency applied, ranging from 300 MHz to 300 GHz, and the microwave power, with ranges between 100 and 900 W, play a fundamental role in the extraction of tannins since their increment, usually, leads to a more efficient recovery [34,35,54]. The time needed for these extraction protocols is short, from 1 to 20 min, achieving a substantial amount of tannins (from 4.11 to 528.5 mg/g of dried extract, Table 2) [43,59,61].

This technique is advantageous since it is quick (uses short extraction times), green (low amount of solvents are required), and efficient (it can provide high recover rates) [39,59]. On the contrary, the equipment for industrial scale is expensive and thermal degradation of tannins caused by microwaves has been described to occur [38]. 

### 3.5. Ultrasound-Assisted Extraction

Ultrasonic cavitation (with frequencies above 20 kHz) is used to create micro-bubbles inside a liquid. These bubbles grow and collapse, causing vibrations and breaking cell walls of plant material. This situation favours a greater penetration of solvents in vegetal tissues [39,65]. Polar ones, such as methanol or ethanol, are considered to obtain higher levels of extraction, ranging from 27.23 to 127 mg/g of dried extract even though when combined with ionic liquid it maximizes its recovery rate up to 630.2 mg/g of dried extract (Table 2) [43,63,65]. Regarding the sonication power, a higher quantity of tannins is extracted with a higher power, but when a superior limit is overcome, chemical decomposition of tannins may occur [38]. In terms of time, this extraction is rapid, employing not more than 30 min protocols. 

The quickness of the extraction, simplicity, and low cost of this ultrasound-assisted method are the main advantages. However, it has been observed that there is no uniformity in the distribution of the intensity of ultrasounds along with the sample [38].

### 3.6. Comparison of Extraction Systems

The main objective of the extraction comparison was to disclose the most efficient method in terms of recovery rates, even though other factors such as cost, time, and use of green solvents were evaluated. Different techniques have been selected to perform the comparison. The variability of the studies, reflected in Table 2, included the use of different vegetal matrixes, different solvents at different concentrations, and different experimental conditions such as very diverse extraction times and very variable instrumental parameters. Considering the variability of conditions, the performance of the comparison has resulted in being very complex. When possible, studies using the sample species but different extraction methods were selected. A summary of these cases is presented in this section. 

In *Lavatera thuringiaca,* few biocompounds were extracted using pressurized water, microwave, and ultrasonic extractions (PWE, ME, and UE, respectively) [58]. The extraction of condensed tannins revealed very similar values for all the extraction techniques 72.23, 71.15, and 71.78 mg/g of dry biomass for PWE ME, and UE, respectively (Table 2). Another study based on *Galla chinensis* employed ME, UE, and the combination of both techniques, using ethanol and 1-butyl-3-methylimidazolium bromide ([bmim] Br) as an ionic liquid to extract tannins [43]. Results obtained for this species showed a general high content of tannins. The lowest recovery rate using UE or ME with ethanol as solvent displayed values of 491.20 and 528.5 mg/g, respectively while the combination of UE and MWE with [bmim] Br lead to the highest value, 630.2 mg/g (Table 2). Finally, tannins are a well-known component of wine, thus numerous studies have been focused on the extraction of tannins from wine by-products, such as grape seeds, pomace, and peels [42,46,50,53,59,60,64]. Different techniques such as solid/liquid extraction (SLE), superfluid extraction (SFE), or ME have been applied for this purpose. Different units have been used to express the tannin’s content. Recovery of tannins from grape by-products when using SLE or ME has been expressed as 12.3 g/L or 22.27 mg/L, thus SLE seems to be a more efficient technique [42,60]. SLE, PWE, and UE have been also treating grape pomace or skin obtaining similar values that ranged from 52.9 to 86.67 mg/g [50,53,64] (Table 2). On the other hand, when using grape seeds as a matrix, recoveries are increased ten times, approximately. In this case, the extraction performed with ME provides 528 mg/g and the one developed with SFE 770 mg/g [46,59]. 

As shown in the previous section, the selection of relevant species and adequate tissues makes the difference in the final recovery rate; nevertheless, it may be maximized by choosing the appropriate technique. In general, SLE has been widely used for the extraction of tannins from very different vegetal matrixes due to its low cost, low difficulty performance, and its traditional application. However, techniques such as SFE, ME, or UE have been demonstrated to be efficient for extracting high amounts of tannins using short experimental times which represent a vantage to avoid the degradation of tannins during the treatment. From these methodologies, the most efficient one might be UE since its relative cost is lower.

## 4. Technological Applications

### 4.1. Coagulants: Environmental Application

The coagulation is essential for the treatment of surface water and industrial wastewater. It is a well-known process that involves the destabilization of colloids and other suspended substances promoting the aggregation as weightier and greater flocs. Usually, the coagulants employed in these processes are inorganic compounds derived from aluminium salts and iron-like such as ferric chloride (FeCl_3_) or aluminium sulphate (Al_2_(SO_4_)_3_) [66]_._ However, researchers have been searching for new and natural sources of coagulants, generally obtained through the plant extracts. Vegetal-based coagulants represent an alternative for these inorganic metal-based ones, characterized for being advantageous since they permit the production of biodegradable sludge, their alkalinity consumption is lower, and can be obtained by renewable sources. Some of the new compounds are tannin-based coagulants and they have been applied in the treatment of different waters showing comparable and sometimes even better performances than the conventional ones [67].

The most studied plant-based tannin used as a coagulant in the treatment of wastewaters has been isolated from *Acacia mearnsii*. There is a commercial tannin-derived coagulant, named Tanfloc SG, consisting of a cationic polymer obtained through the chemical modification of natural tannins extracted from *Acacia mearnsii.* Along with scientific literature, many works compare the performance of synthetic tannins against natural ones, while some others have optimized the experimental conditions to achieve the maximum throughput and then have applied them to natural samples. Different types of effluent waters have been treated using natural-based coagulants, even those with high loads of antibiotic-resistant bacteria (ARB) and antibiotics. The presence of these microorganisms and these drugs in water displays the wide-world issue that the excessive consumption of antibiotics has generated and that are a threat to the environment and public health. Few works have compared the performance of tannins from *Acacia mearnsii* (natural ones or those commercially modified, Tanfloc SG) versus aluminium-based coagulants, such as Al_2_(SO_4_)_3_ to suggest a cost-effective technology for treating final wastewaters. One study used *Acacia mearnsii*-based coagulants for treating urban effluents. Obtained results displayed that, even though Al_2_(SO_4_)_3_ showed better performance, both the synthetic and natural-based ones were able to decrease turbidity, the colour of secondary treated urban wastewater. Additionally, both coagulants were capable of reducing the load of prokaryotic organisms up to two log units just after the treatment of the water [68]. In another study, tannins from *Acacia mearnsii* were tested for treating wastewater from dairy industries. Data showed no significant statistical differences when evaluating colour, turbidity, chemical oxygen demand (COD), or total solids (TS) removal. *Acacia mearnsii* tannins presented lower alkalinity consumption than the aluminium-based coagulant, interfered less in electrical conductivity, and possessed a wider pH range of utilization. In addition, the flocs formed while using tannins presented better stability accompanied by a reduced breakage achieved through the application of slow mixing time. Lastly, the lower ash content, the higher amount of volatile solids, and fixed carbon of the final sludge [69]. Similarly, another research evaluated the capacity of a coagulation–flocculation–sedimentation procedure based on the use of Tanfloc SG against an iron salt for their application in the treatment of dye-house effluents. Iron sulphate showed very promising features in acid conditions; however, neutral and alkaline conditions are more convenient for this study since effluent pH is usually around 8. Under these parameters, the tannin coagulant was much more efficient. It achieved a total discoloration at a concentration of 180 mg/L while higher doses of iron sulphate (up to 240 mg/L) displayed a poor de-colorization (20%) and flocs settleability [70]. The lack of significant differences among coagulants in all the cases permits establishing that tannins from *Acacia mearnsii* tannins provide a green solution for the treatment of coloured and turbid effluents generated from very different wastewaters as those produced in urban areas, dye-houses, or dairy industries [66,67,68].

The use of natural tannins has also been optimized by their mix with other reagents. This is the case of tannins extracted from *Acacia mearnsii* de Wild that were combined with NH_4_Cl and formaldehyde for water purification and wastewater remediation. This mixture was confirmed to be a useful tool for avoiding surfactant and dye-pollution from wastewater. The variable parameters for the cationic coagulant were temperature and the tannin–NH_4_Cl ratio, being the latter more influential than the first one. Optimal parameters found were established at 2 g/g using 25 °C for dye removal and 36 °C for surfactant elimination. Finally, the last step of this setup was to apply a combined and optimized coagulant in natural media. This final approach was tested in dye, surfactant, surface river water, and municipal wastewater, confirming the experimental results [71].

In some studies [72] tannins were provided by Water Chem Pte Ltd and showed to be effective for the post-treatment of leachates. Tannins de-colorization effectiveness of biologically treated landfill leachate was evaluated through a coagulation–flocculation assay. Optimal results were obtained at a leachate pH of 5, using an experimental time of 3 and 10 min for the flocculation and sedimentation, respectively. These parameters were demonstrated to perform the highest colour removal (82%) at a coagulant concentration of 100 mg/L combined with 1 mg/L anionic polyacrylamide.

### 4.2. Adhesives: Wood, Tires, Concrete

Historically, a different type of natural adhesives, based on soy protein, lignin, starch, and tannins have been utilized. Tannin applications have been around for a long time. A typical reaction used for triggering their polymerization is based on a mixture with formaldehyde that reacts with tannins. The most reactive positions of the flavonoids are commonly placed at the A-ring where methylene groups allow crosslinking which builds the main scaffolds for achieving tannin-based adhesives. This formaldehyde-mediated reaction occurs similarly for other adhesives even though it is not so extensive. Phloroglucinol-like A-rings of procyanidin-type tannins, such as the ones present in pine bark tannins, have shown reactivity with formaldehyde identical to the phloroglucinol ones [73].

The required amount of formaldehyde for obtaining a resistant and strong tannin-based adhesive represents about 10% of the quantity used for its equivalent synthetic adhesive. However, formaldehyde has been recognized to possess high systemic toxicity and it has been included as a CMR (carcinogenic, mutagenic, or toxic to reproduction) substance and classified in the category of carcinogens 1B. Thus, there is an increasing concern to diminish or even eliminate it [74]. Therefore, several studies have tried to replace the use of formaldehyde in wood adhesives. The tannin–glyoxalate Kraft lignin (TGKL) resin is one of these new successful substitutes but it is not the best option for using in wet environments since its main disadvantage is its low water resistance. To solve this issue, researchers suggested increasing the degree of modification of this TGKL using a hyperbranched poly-amine-ester (HBPAE) to synthesize a new green adhesive named MTGKL. Then, a comparative study for TGKL, MTGKL, and a commercial phenol–formaldehyde (CPF) resin was performed using different techniques such as FTIR, ^1^H and ^13^C NMR spectroscopy. Results obtained determined that the poly-amine-ester-based modification of TGKL to get the MTGKL improved its water-resistance avoiding the appearance of lamina when soaking with cool water over plywood treated with MTGKL. Additionally, tensile strength values for dry (40 MPa) and wet (28 MPa) MTGKL are higher than those for TGKL and CPF resins [75].

Different compounds can be added to tannins to develop a tannin adhesive free of formaldehyde that was demonstrated by mixing tannins with very definite proportions of furfuryl alcohol and glyoxal in water. The final protocol lasted less than 2 h and it includes two main steps: Pre-condensation (60 min, at room temperature under acid conditions) to synthesize the pre-polymer and the final polymerization (pre-polymer was mixed with mimosa tannin at 70 °C for 30 min). Both products of these reactions were analyzed by 13C NMR and MALDI TOF. Results confirmed the reaction between furfuryl alcohol and glyoxal, and that the features of the tannin–furfuryl–glyoxal adhesive (TFG) are due to the crosslinking mediated through hydroxymethyl groups. TFG was observed to improve its hydrophobic properties when cross-linked with a low percentage of epoxy-resin (12%). Final products obtained along these experiments were also demonstrated to present better elasticity than its formaldehyde-based analogues [76].

The reinforcement of tires is due to a set of cords that are responsible for the strength. For the tire industry it is essential to prevent blowout risks, thus it is needed to control deformation tires but also offer the comfort of riding, properties which are mainly owned by the tire rubber adhesion. Some tannin adhesives that have been successfully tested include tannin resorcinol–formaldehyde, thermosetting tannins formulation, and also cold setting tannins adhesives [11,51]. However, as the current trend tends to avoid the use of formaldehyde many studies have been focused on its replacement. It has been demonstrated that condensed tannins can substitute the majority or even all of the resorcinol–formaldehyde–latex (RFL) dip, especially when using polyester cords. As an example, an extract obtained from the middle septum tissue (pith) of pecan nuts has been proved to be the perfect substitute for resorcinol adhesive, since its use allows to double the tire cord pull-out force when compared with the standard dip [77]. Other sources of condensed tannins used to replace the standard dip besides the pecan nut pith are the bark of southern pine trees or peanut skins. These replacements can equalize the tire cord adhesion test (TCAT) geometry obtained with the standard dip when the cords are made of nylon embedded in a typical styrene-butadiene rubber (SBR) vulcanization and when bounding polyester cord, they showed pull-out forces substantially higher than the standard RFL dip [78,79].

The phenolic nature of tannins has allowed increasing their application fields. Lately, tannins have been tested as superplasticizers in cement technology. Initially, plasticizers were used as dispersion agents to incorporate concrete preparations. The addition of these compounds avoids using excessive amounts of water in the final product which results in a product with better workability and enhances its strength development. Unfortunately, while plasticizers improve these properties, they lengthen the setting and in the strength during the early development of concrete. Superplasticizers represent the alternative since used in small amounts they strongly contribute to concrete fluidity without compromising or retarding its setting. Apart from heavily sulfonated melamine–formaldehyde resins, tannins are the only other superplasticizers which have been applied for cement technology [11]. Tannin extracts from quebracho, mimosa, and pine are all examples of good cement superplasticizers. Those from mimosa and pine show better performance, since they have a noticeable effect on fluidification of the cement, with very low dosages, between 0.25% and 0.5% [80]. The addition of tannins provides cement workability enhancement without requiring the incorporation of extra water which avoids the retard of setting and the loss of strength in the final or initial form. When using tannins as superplasticizers, slumps of 200 mm can be achieved with no water content increase or by reducing it up to 30% [80]. Therefore, the behaviour of the tannin extracts as a cement superplasticizer is due to the balance of different factors and properties. The presence of hydroxyl groups, in the B-rings of the flavonoid units, allows complex metallic ions such as the ferric, ferrous, or aluminium ones which confer them the capability of condensing themselves. The presence of silicate and aluminium components of cement triggers this self-condensation reaction which leads to the formation of tannin complexes and therefore to its increase in the molecular weight. Their solubility improves when they get sulphonated, in this process the heterocyclic pyran ring gets opened and introduces the sulphonic group at the C2 sites of some of the flavonoid units [81]. Lastly, the stabilization of their molecular weight induced by the addition of urea, results in their self-condensation that decreases the association with water in colloidal tannin extracts. 

### 4.3. Ore Flotation Agents

Flotation is a technique applied to industrial processing that allows separating materials, mostly minerals, by modifying the hydrophobicity of their surfaces to enhance or suppress their attraction for water or polar solvents. It is a process widely used in metallurgy to concentrate ores. For this application, the ore is ground until a powder is obtained after it is mixed with an aqueous solution of different chemicals that possess an active surface. Then, the mixture is vigorously aerated with the consequent formation of bubbles that trap mineral fragments and bring them to the foamy surface, which is later removed. 

Among the known tannin properties, one of them is their ability to form complexes with metal ions such as iron, copper, or noble metal salts. This feature makes them interesting for use as a selective modifying agent in flotation due to their functions, depressant, and dispersant. Additionally, this type of compound allows replacing highly toxic compounds used as flotation agents by a material of plant origin. Thus, for decades, tannins have been used as depressants in flotation processes and applied to improve the efficiency of extraction or concentration of gold from complex ores, jamenosite, strontium, rare earth oxides, scheelite, fluorite, and sulphides (copper-lead-zinc-nickel ores) [82,83,84]. Many studies have been performed to evaluate the capacity of different types of tannins, as well as to optimize the parameters that maximize the extraction of target metals. 

Tannins are capable of carrying out selective adsorption on the surface of some sulphide minerals such as stibnite, arsenopyrite, and chalcopyrite, as well as exerting a selective effect on the adsorption of a sulfhydryl collector which makes them interesting compounds for effective recovery of minerals such as those forming complex gold ores [82,83,84]. Antimony and gold are two metals of recognized commercial importance. They are naturally found as a complex of gold-antimony ores. Their separation involves difficulties since their content is very low, their distribution along the ore is irregular, and gold usually appears concentrated with iron sulphides, including arsenopyrite. The processing of these ores yields gold-bearing, gold-sulphides, and antimony. Stibnite or antimony sulphide and arsenopyrite can be barely separated since differences of their flotation properties are minimal in the presence of most of the floatation agents. To solve this issue, a study was developed using tannins due to their hydrophilic and binding properties to the collector compounds and the free areas of the mineral surface. Tannin added at rates between 100 and 200 g/t and in the presence of compound collectors permit stibnite to maintain a constant flotation rate at 70% while arsenopyrite floatation is reduced to 15%–20%. Thus, tannins are capable of suppressing arsenopyrite and provide active flotation of antimony sulphide; but do not form a dense covering on stibin and do not desorb copper dithiocarbamate from the surface of the activated mineral. However, by adjusting the consumption rate of phytogenous, the modifier-tannin controlling flotation activity of stibnite and arsenopyrite is possible [83]. Nowadays, another important source of gold is recovered from e-wastes (wastes from electric or electronic devices) which have been considered as secondary gold ore. The rising production of e-wastes has triggered the optimization of recovery processes to obtain the highest amounts of gold. Different approaches have been evaluated using tannins, especially those obtained from persimmon and bayberry. Tannin-based resins were able to recover gold through its reduction from the auric (Au III) to the elemental form. This technique has been demonstrated to be selective for gold recovery avoiding the uptake of other minerals such as iron, zinc, copper, lead, or nickel, etc. The use of tannin-based resins provided recovery rates of 642–1799 mg/g which were higher than those obtained from resins chemically modified with amines (1320 mg/g, on average) [85]. 

In a study focused on jamesonite, five tannin extracts obtained from different tree species (*Acacia mangium*, bayberry, emblic, larch, and valonia) were observed to enhance the flotation of this metal. Even though all of them showed an improvement on flotation, larch tannin extract was the best of them. Additionally, a trial performed at industrial level, in the same work, concluded that the addition of larch tannins at a concentration of 20 g/t increased the content of antimony from 13.1% to 17.6% and lead from 14.8% to 19.9%, in the final concentrate. Its application also decreased the degree of zinc from 9.1% to 6.5%, and allowed obtaining a recovery of antimony and lead of 84.5% [86]. 

Tannins were also tested to separate scheelite and calcite, but in this case it has been proved that the best option for achieving that is acidified water glass as depressants of calcite without imposing obvious adverse effects on scheelite flotation [87].

Tannins extracted from quebracho (*Schinopsis balansae*) have been widely used in the industry as depressing agents. As tannins obtained from other species, some of the main uses of these tannins include their application for obtaining differential flotation rates of copper, lead and zinc circuits, or for the precipitation of germanium from zinc ores, or for the recovery of ultrafine gold [88]. However, it is relevant to its application for the separation of fluorite from calcite, which is critical since both minerals share a common cation, calcium [88]. As a depressant, it can be underlined that its use in pyrite as a depressing agent improves the copper/iron (Cu/Fe) ratio. Most of the global production of copper is usually extracted from sulphide ores, where copper represents about 1%. From these numbers, it is clear that this process implies huge expenses in terms of the amount of mined rock, energy, and water waste, apart from the environmental impact derived from the use of toxic compounds, among others. Therefore, any implement in the process of copper extraction means an important saving of resources. A study evaluated the repercussion of modifying different parameters, including different pH values and the addition of diverse concentrations of few quebracho tannins, to optimize this process. The best conditions for disseminated copper ores were developed in the alkaline range. Moreover, the depressing effect of tannins on pyrite increases the Cu/Fe ratio in concentrates between 8% and 40%. All these vantages have allowed concluding that quebracho tannins can be a cleaner alternative to conventional depressing reagents in sulphide flotation [89].

Another study allowed demonstrating that the tannins extracted from oak bark are capable of forming stable insoluble compounds with Fe (III) on the surface of pyrrhotite and arsenopyrite. This allows the hydrolyzing of the mineral surface to improve and decrease its buoyancy in floating conditions [90].

### 4.4. Fabric Manufacture

#### 4.4.1. Leather Industry

Leather is an organic material made of protein fibres, mainly collagen, that may break down over time, reducing its durability and proportionally its economic value, affecting directly to leather goods. This issue has been solved with a process called tanning that prevents the decomposition of skin and makes it inalterability and resistance. Tannins can bind to proteins and stabilize their structure. The main component of skin, collagen, in the presence of about 15% to 40% of tannins is stabilized and proving absorbent and breathable properties to the treated tissues. Tanning is a millenary methodology, considered to be one of the oldest processes used to treat leather; that already existed in northwestern regions of Europe after the Roman conquest. This technique, known as vegetable tanning, since it uses exclusively tannins along the full process, was applied until the end of the 19th century. At that moment, a new technique called chrome tanning, based on the combination of mineral salts (chromium (III) salts) and vegetable tannins just applied at some stages of the process commenced being more employed. The historical application of tanning implies many objects along the years thus conservators are very concerned about the use and restoration of tanned leather. Colours obtained with hydrolysable tannins are lighter than those obtained with condensed tannins. When leather is treated using these latter ones, it is considered to be less effective in terms of protection since it more easily adsorbs pollutants that may trigger the collagen hydrolysis. Therefore, the selection of adequate condensed tannins for treating leather aimed at conservation and restoration purposes is crucial. Therefore, it is necessary to evaluate different sources of condensed tannins that allow achieving high quality tanned leather since conservators claimed that leathers tanned with hydrolysable tannins cause further restoration expenses [11,91,92,93]. The use of natural tannins is limited by their sensitivity to photo-oxidation, limiting their applications. After prolonged exposure with UV light, there is a change in the colour of the leather that is mainly due to two factors. One of them is the darkening reaction of the leather due to the formation of quinones on the phenolic structure of the tannin. The other one is the leather-lightening reaction due to the photodegradation of the system [94]. Nevertheless, when photooxidation represents a problem it can be reduced by the condensation of tannins with sulfonated synthetic aminoplastic resins. An example of these synthetic resins is melamine–urea–formaldehyde (MUF) which provides softness and flexibility to leather. However, it contains free formaldehyde and low tanning capacity due to its low astringency, which can be counteracted with the use of polyphenols with no need for chromium salts [11]. 

An example of this application is aqueous extracts with small amounts of ethanol of *Pinus pinaster* bark which can be used as an eco-friendly tanning agent that can partially replace chromium salts. Therefore, it will reduce some of the environmental impacts typically associated with the leather industry [41]. This can also be obtained from *Eucalyptus globulus* bark, being the best conditions of extraction water, 140 °C and 120 min, and obtaining, as a result, an extract with leather retaining aptitude equivalent to a commercial extract of chestnut. Its application revealed also good performance in the production of leather articles such as box-calf and nubuck [95]. 

#### 4.4.2. Dyeing Industry of Natural Fibres: Cotton, Wool, Silk

The use of tannins as dyers of natural fibres such as cotton or silk has been done for hundreds of years in combination with metal salt (e.g., CuSO_4_) which are used as mordants, it is a colour fixer. Nowadays, tannins are also employed in fabric manufacturers for the production of clothes based on tissues such as cotton, wool, or silk [96]. Dye using tannins is achieved by a continuous and very rapid process which consists of passing the fabric through a small padding trough followed by intensive squeezing between expression rollers, then dye fixation by hot air while drying and finally the samples are thoroughly rinsed and air-dried. This technique, called pad-dry dyeing, can be applied with tannins extracted from eucalyptus leaves where they can be abundantly found [96]. Different works have studied how to improve the staining capacity of tannins. The addition of protein isolates to tannins obtained from the bark of *Xylocarpus granatum* increased the colour strength according to protein and dye concentration and dyeing time and temperature. The improvement of colour absorption observed after treatment with proteins was due to the formation of insoluble complexes between proteins and tannins mainly owed to the formation of hydrogen bonds and hydrophobic interactions [97]. Different sources of tannins have been tested for using them as dyers. A study evaluated the combination of *Punica granatum* peel and walnut shell with diverse types of mordants. Changes in colour properties were observed depending on the mordant employed. The study showed that pre-treatment with metal mordants substantially improved the dyeing and solidity properties of wool fabrics. Additionally, tannins presented antibacterial properties that were enhanced with the use of metal salts (e.g., copper, tin, and aluminium salts). This antimicrobial activity makes the peel extracts of *Punica granatum* and walnut shell interesting antibacterial agents for hospital textiles, as well as its application in sports or domestic clothing to avoid bad odours [98]. This antibacterial property was also found in extracts rich in tannins obtained from *Emblica officinalis* G. Tannins obtained from dried fruits can be applied as mordant themselves or they can be used in combination with metal-based mordants. Antibacterial properties when using tannins from *Emblica officinalis* G fruit were similar to those of *Punica granatum*. The use of mordants gave a better intensity of colour, washing, and lightfastness. Of the mordants studied, the best option was to apply a 0.5% and 1% copper sulphate since its antimicrobial activity was maintained even after 20 washes for both cotton and silk natural dyed fabrics [99]. These were not the only studies carried out to develop bioactive fibres that could be used in textiles related to the medical field. Extracts obtained from the *Acacia nilotica* bark were evaluated in terms of potential for coloration, antioxidant, and toxicological activities. Fabrics can be quickly dyed with enhanced colours by using several different metal salts, such as alum, ferrous sulphate, and stannous chloride. Nonetheless, the addition of these mordants have shown a reduction on the antioxidant activity of dyed woolen yarn than unmordanted samples, but it showed no toxicity. Optimum conditions for dyeing were obtained when a dye concentration of 10% was applied at pH 3, for 90 min at 50 °C [100]. Another interesting property of tannins is a flame retardant for silk textiles. Condensed tannins obtained from *Dioscorea cirrhosa* were easily adhered in weakly acidic conditions to silk. Retarding flame features of fabric tanned with *D. cirrhosa* was demonstrated by limiting oxygen index, vertical burning, and pyrolysis combustion flow calorimetry tests. This work also displayed the antibacterial and antioxidant properties that implied the use of *D. cirrhosa* tannins [101].

### 4.5. Food Additives

Naturally, tannins are part of the diet since they are present in many plant matrices that we eat. The antioxidant properties of tannins are of great relevance and importance in food applications, due to their ability to prevent disorders related to oxidative stress, such as cardiovascular diseases or cancer [102,103]. However, the characteristic bitter taste produced by tannins makes them organoleptically deficient additives, so, currently, their main use as an additive is reduced to the feeding of livestock and animals, which produces great benefits.

It has been found that the addition of tannins obtained from quebracho in diets of lambs and sheep decreases the oxidation state of muscle, liver, and plasma, as well as improves the colour stability of the meat since it slows down the oxidation process of the myoglobin during storage [104]. On the other hand, it is necessary to consider the stability and bioavailability of tannins when crossing the gastrointestinal tract. Hydrolysable tannins are degraded during this step, before their absorption, while condensed tannins are not degraded or hydrolyzed along this tract, so they cannot be absorbed either [105]. Therefore, taking into account the increased antioxidant activity in animals fed with tannins, it is speculated that the supply of these compounds may reduce the expenditure of other systemic antioxidants, while they may protect additional food nutrients, preventing its oxidation during the digestive process and promoting their intact arrival to the circulatory system [104,106].

Scientific literature and reports point to the antibiotic resistance that microorganisms have acquired over the last decades, it is mostly due to the excessive administration of these drugs. Fortunately, veterinary regulations have been published to limit and reduce the addition of antibiotics to animal feed [107]. As an alternative for the use of traditional antibiotic substances, a possible solution is the addition of natural compounds obtained from plants, such as tannins, with demonstrated antimicrobial activity [108]. It has been proven that the complementation of animal food, specifically ruminants, with tannins provides such interesting and favourable advantages as antibacterial, antifungal, antioxidant, anthelmintic, antidiarrheal, and antihemorrhagic properties, as well as the reduction of acidosis and methane production and the increase in meat production [77,109,110].

Regarding the legal frame regulating the use of tannins as an additive in animal feed, the available information is limited, to our knowledge. Little information legislating tannins has been published under the Commission Implementing Regulation (EU) 2017/66 of 14 December, 2016, that concerns the authorization of tannic acid as a feed additive for all animal species, and it states that the recommended maximum content of tannic acid shall be 15 mg/kg of complete feeding stuff [111,112].

In human food, in the market, there are tannin preparations for incorporation in industrial preparations. However, and despite not having much information about it [113], the use of some of them is regulated by the Commission Implementing Regulation (EU) No 872/2012 of 1 October, 2012 which Annex I includes certain tannins which are classified as flavouring additives, such as tannic acid or gallic acid, so their food use is allowed [114].

### 4.6. Medical, Pharmaceutical, and Veterinary Applications 

Tannins, as secondary metabolites of plants, participate in their chemical defence, protecting them from possible attacks by pathogens, insects, herbivores, opportunists, etc., as well as adverse abiotic conditions. That is why these compounds show biological activities that can be useful in medical, pharmaceutical, and/or veterinary applications [109]. The main bioactivities described for tannins are detailed below and are the following: Antioxidant, antimicrobial, anthelmintic, antiviral, and anti-inflammatory.

#### 4.6.1. Antioxidant 

Many studies relate phenolic compounds to a powerful antioxidant activity, so tannins are also included in these characteristics. It has been proven that those tannins of greater molecular weight and with a greater amount of hydroxyl groups in their structure are those that achieve better antioxidant actions [115], while the formation of tannin–protein complexes reduces this antioxidant ability [116]. The degree of polymerization, highly related to the molecular weight of tannins, is another characteristic that influences antioxidant properties. It has been proven that those tannins with a higher level of polymerization and more hydroxyl groups in their structure have greater antioxidant properties than simple tannins, thanks to its increased ability to be oxidized. Regarding condensed tannins, a positive and proportional relationship between the degree of polymerization and antioxidant power has been reported; although this relationship is only corroborated in a range of degree of polymerization lower than 10. From that value, the antioxidant capacity of the molecules decreases [117]. However, and despite the research carried out in vitro and in vivo, the mechanism of action that tannins exert on animal tissues is still unknown.

#### 4.6.2. Antimicrobial 

The antimicrobial activity of tannins is due to the toxicity they produced in bacteria, fungi, and yeasts. Although the ability of tannins to precipitate proteins is widely known and demonstrated, the main mechanism of action is based on molecular inhibitions that mostly occur in the cell membrane of microorganisms, such as the inhibition of the formation of complexes that maintain its integrity, generating malformations and increasing their permeability. Other mechanisms are associated with the inhibition of extracellular microbial enzymes, the decrease of the compounds necessary for cell growth, or modulation of metabolism through the inhibition of oxidative phosphorylation capacity or metal ions deprivation [118]. In the case of Gram-negative bacteria, the external membrane they possess increases their protection against tannins action, it has been demonstrated that tannins produce lower toxicity on them. Nevertheless, tannins, especially phlorotannins and condensed tannins, exert antibacterial activity on certain genera and species of that group, such as *Salmonella*, *Escherichia coli* O157:H7, *Shigella*, *Pseudomonas*, *Staphylococcus*, and *Helicobacter pylori* [118,119,120]. It is relevant to note the strong antimicrobial activity that condensed tannins extracted from *Dalea purpurea* and isolated phlorotannins from brown seaweed *Ascophyllum nodosum* displayed against *E.coli* [121]. A higher number of hydroxyl groups seem to be related to a stronger antimicrobial capacity. Flavonols with trihydroxy B ring, such as gallocatechin, produce greater inhibition of bacteria such as *Clostridium botulinum* than those with a dihydroxy B ring, such as catechin [122]. That is also the case of the phlorotannins, which possesses a high ratio of hydroxyl substitutions and they have been proven to possess a stronger antimicrobial capacity than condensed or hydrolysable tannins [121]. This feature may be due to their ability to release peroxide of hydrogen when they are oxidized [123]. 

#### 4.6.3. Anthelmintic 

The activity of tannins as anthelmintics is known to be greatly influenced by the structure and chemical composition of these natural compounds, the parasitic and the host species [124,125]. However, it has been demonstrated that tannins produce a direct mechanism in parasite cells at different stages of the life cycle, causing reductions in invasive capacity in the host, the development of eggs in the third larval phase, and the excretion of eggs by an adult part [125,126,127], as well as an indirect action, due to an increasing host resistance to the invasion [128]. These antiparasitic properties have been proved in both in vitro and in vivo studies, being successful against species such as *Haemonchus contortus* and *Trichostrongylus colubriformis*, and some other species of nematodes as *Cooperia, Ostertagia, Oesophagostomum,* and *Strongyloides* [129,130]. The analyzed tannins that showed bioactivity were extracted from plants such as sainfoin (*Onobrychis viciifolia*), trefoil (*Lotus pedunculatus*), green tea (*Camellia sinensis*), or quebracho (*Schinopsis balansae*) [129,131], among others, and it was shown that they exerted a dose-dependent action. Finally, the animals that are most commonly used for in vivo tests are sheep and cattle [132,133,134].

#### 4.6.4. Antiviral

There are different mechanisms of action involved in the development of the antiviral capacity of tannins. Some hydrolysable ones, such as the chebulagic acid, exert a considerably potent antiviral action in vitro, which translates into a reduction in mortality and an improvement in symptoms in tests performed on animal models [135]. This action is caused by the fact that those tannins avoid the viral replication through an inhibitory action based on their activity over certain transcriptases, proteases, and integrases [136], while other tannins, namely punicalin and geraniin, inhibit the covalent closed circular DNA (cccDNA) production or cause protein aggregation. Phlorotannins, such as those obtained from *Eisenia bicyclis* or *Ecklonia cava*, also have antiviral action, since they inhibit virus entry and/or inhibit replication, an action that epigallocatechin also achieves against hepatitis C virus, which is abundantly present in green tea [137]. Some of the viruses that are affected by the presence of tannins are the human papillomavirus, the human immunodeficiency virus, hepatitis B virus, influenza virus and some adenoviruses, enteroviruses and noroviruses, etc. [138,139]. As in the case of other bioactivities, the mechanism of action and the antiviral effectiveness that each tannin exerts is greatly influenced by its structure and chemical composition [140].

#### 4.6.5. Anti-Inflammatory 

This biological function is highly related to the antioxidant activity of tannins, especially regarding hydrolysable tannins, since some of the speculated mechanisms of action have a direct association with the radical scavenging capacity of these compounds [141]. On the other hand, condensed tannins and phlorotannins have been highlighted as inhibitors of the formation of nitric oxide, a pro-inflammatory molecule. Phlorotannins, such as those obtained from *A. nodosum* and *E. cava*, have been also suggested to decrease the production of cytokines and prostaglandin-E2 [142,143], molecules of great interest due to their involvement in inflammation-mediated diseases, such as obesity or diabetes. Regarding hydrolysable tannins, those extracted from *Myricaria bracteata* achieved a very significant anti-inflammatory activity in ear edema and arthritis induced in mice, using croton oil and collagen, respectively [144].

The European Medicine Agency (EMA) contemplates the aforementioned bioactivities of tannins and allows the use of extracts containing the above-cited compounds for therapeutic purposes in humans and animals. However, the EMA calls on researchers to develop further studies on this field to complete a full picture of these compounds. In this context, the bioavailability of tannins has to be considered and analyzed, especially for oral administrations. Hydrolysable tannins have been described to be degraded along the gastrointestinal tract before they can be absorbed; while intestinal conditions seem to not alter condensed tannins hindering their absorption. Thus, it is necessary to disclose the mechanism of action that explains how intact tannins or they degradation sub-products act at tissue or cell levels [109].

Contrary to that, the Committee for Veterinary Medicinal Products reported that the toxicity produced by oral administration of tannin is low (LD_50_ = 2250–6000 mg/kg), but it increases greatly when administered intravenously (LD_50_ = 80 mg/kg) [145].

Information regarding current legislation about tannins use in medical and/or pharmaceutical preparations is scarce. Tannins, as a set of compounds, are classified according to the “new statistical classification of products by activities (CPA)”, detailed in Regulation (CE) No. 451/2008 of the European Parliament and of the Council, of April 23, 2008, which repeals the Council Regulation (EEC) No. 3696/93 [146], as products with agriculture, livestock, forestry, and fisheries applications. Specifically, they have been classified under the titles “tanning or dyeing extracts; tannins and their derivatives; colouring matter not classified elsewhere” and “tanning extracts of plant origin; tannins and their salts, ethers, esters and other derivatives; colouring matter of vegetable or animal origin”, with the CPA codes 20.12.2 and 20.12.22, respectively. 

## 5. Conclusions

Along with history, tannins have represented very useful compounds in wood and fabric industries. In the last decades, the multiple chemical properties of tannins have revealed them as a promising alternative to reduce the use of metals or other chemical substances applied as coagulants or as tannin modifiers and that have been disclosed as a threat for human health and harmful for the environment. Tannins are natural molecules that can be obtained from several and diverse vegetables. Some of the most representative species synthesizing or bio-accumulating a high amount of tannins considered along this review were *Terminalia*, *Betula* and *Acacia*. Regarding the extraction methods, nowadays, the traditional solid/liquid extraction is the most used technique in the industry, but the perspective is to apply techniques that are more environmentally friendly. Among all the different extractions, some authors have considered microwave, ultrasonic and, especially, pressurized water extraction as the most promising techniques. 

The diversity of chemical structures encompassed in the tannins family has allowed their use from multiple approaches. These vegetal-based compounds represent an alternative for substituting the use of inorganic metal- or chemical-based coagulants.

Tannins have an important role in the development of new eco-friendly materials that can be further applied as coagulants, adhesives, superplasticizers, or floatation agents. The most relevant tannin-based coagulants come from *Acacia mearnsii* and permit to obtain biodegradable sludge after the treatment, reducing the impact on the environment. Same *Acacia mearnsii* tannins, besides other ones, such as those extracted from chestnut wastes and pecan nut pith, have been tested to replace the use of formaldehyde, classified as a carcinogen, and successful adhesives have been polymerized. In fact, in the specific field of tires, it has been demonstrated that condensed tannins can substitute the majority or even all of the resorcinol–formaldehyde–latex dip. Tannins are also an alternative to the only other known superplasticizers used for concrete, the sulfonated melamine–formaldehyde resins. In the field of mining, tannins have been demonstrated to be a very useful tool to selectively separate and concentrate compounds found scarcely in complex mixtures of material. Additionally, tannins are characterized to be a more environment-friendly option than those based on chemical products.

Tannins result in a very useful group of ingredients to allow improving colour strength while enhancing fabric characteristics contributing to antibacterial, antioxidant, and flaming retardant properties. Additionally, tannins use is advantageous since they are natural dyes and have a better opinion among consumers. Therefore, all these features reveal tannins as a sustainable and eco-friendly source of dyes that permit to revalue fabrics. 

The polyphenolic nature of tannins reveals them as an important source of therapeutic molecules. Many studies have shown the antioxidant, antimicrobial, anthelmintic, antiviral, and anti-inflammatory activities of diverse tannins differently administrated, even included as a food additive. The variable response that these compounds have been shown was to be highly dependent on the applied dose and the synergies established between molecules. Nevertheless, these studies have allowed disclosing many aspects of their structure–activity relationships, metabolism, etc., even though deeper studies are required to understand their mechanism of action among other undetermined factors. Therefore, it is necessary to keep researching, so that a favourable benefit/harm balance is achieved and so they may be increasingly used in pharmaceutical formulations to promote their medical and veterinary use.

Therefore, this review aimed to provide a detailed overview of the most relevant organisms in terms of tannins content, the most efficient, fast and eco-friendly extraction methods, and the multiple industrial applications of these isolated compounds to very different fields such as materials, fabrics, food, or medicine. 

## Figures and Tables

**Table 1:** Major sources of tannins. Species and their target tissues considered as a source of both hydrolysable and condensed tannin types by the determination of the concentration of these molecules using different detection methods and expressed in mg/g of dry weight (dw), except when other units were indicated.

Species	Tissue	Type	Method	Concentration (*mg/g dw*)	Reference
*Carya illinoinensis*	Nut	H and C	HPLC	0.22 ± 0.11 H; 4.66 ± 2.28 C	[2,16]
	Kernel (K) and nutshell (N)	C	Vanillin-HCl	0.4–5.3 C (K); 0.5–876 C (N)	[17]
*Juglans regia*	Nut	H and C	HPLC	0.05 ± 0.01 H; 0.06 ± 0.03 C	[2,16]
*Ribes nigrum*	Juice and extracts	H and C	ST	0.04 ± 0.01 H; 1.14 ± 0.50 C	[2,26]
*Fragaria* sp.	Fruit	H and C	HPLC	0.77 ± 0.006 H; 0.99 ± 0.08 C	[2,16]
*Rubus fruticosus*	Fruit	H and C	HPLC	2.10 ± 0.60 H; 0.25 ± 0.20 C	[2,16]
*Rubus occidentalis*	Fruit	H and C	HPLC	2.43 ± 0.83 H; 0.38 ± 0.35 C	[2,16]
*Punica granatum*	Fruit, seeds, juice (J)	H	HPLC	0.12 ± 0.06 H; 7–1169 mg/L (J)	[2,27]
*Psidium* spp.	Fruit	H	HPLC, ST	2.35–55.5 H	[2,28,29]
*Mangifera indica*	Fruit	H and C	ST	0.55–0.95 H	[2,30]
*Prunus dulcis*	Fruit	H	HPLC	0.27 ± 0.07 H; 1.62 ± 0.95 C	[2,31]
*Desmodium ovalifolium*	Leaves and twigs	C	RDABA	57–273 C	[19,20]
*Gliricidia sepium*	Leaves	C	RDABA	25–186 C	[19,20]
*Manihot esculenta*	Leaves and stems	C	RDABA	26–169 C	[19,20]
*Arachis pintoi*	Leaves and twigs	C	RDABA	40–186 C	[19,20]
*Castanea sativa*	Fruits and bark	H and C	HPLC	0.7–89 H; 0.0001–167 H and C	[2,21,22]
*Terminalia* sp.	Fruits	H and C	HPLC	126–822 H and C	[23]
*Quercus*	Wood	H	HPLC, GC	19.26–47.26 H *Q. robur*; 11.55–30.88 H *Q. petraea;* 8.18 ± 0.18 H *Q. alba*.	[24,25]
*Betula* spp.	Leaves	H and C	HPLC	0.72–60.67 HT; 47–103C	[32,33]
*Pinus sylvestris*	Needles	C	HPLC	70–80 C	[34]
*Eucalyptus globulus*	Leaves	H and C	HPLC	93.57 H + Phloroglucinol	[35]
*Acacia* sp.	Leaves	H and C	ST	*185* H and C *A. angustissima;* *84* H and C *A. drepanolobium,* *256* H and C *A. nilotica;* *98* H and C *A. polyacantha;* *217* H and C *A. tortilisand;* 226 H and C *A. Senegal*	[36]
*A. mearnsii*	Bark	C	Butanol-HCl	235 *C*	[37]
			HPLC	108 C	
*Caesalpinia spinosa*	Pod	H and C	Butanol-HCl	4.6 C	[37]
			HPLC	647 H	
*Schinopsis lorentzii*	Heartwood	C	Butanol-HCl	123 C	[37]
			HPLC	164 C	

Tannin types: Hydrolysable and condensed (H and C), hydrolysable (H), condensed (C). High-performance liquid chromatography (HPLC), gas chromatography (GC), spectrophotometric techniques (ST), radial diffusion, and acid butanol (spectrophotometric) assays (RDABA).

**Table 2:** Extraction methods for scaling up to the industry use of tannins. Different vegetal matrixes, considered tannin sources, have been tested using diverse extraction techniques combined with different experimental conditions (mostly based on aqueous (aq) dilutions), have been applied to different vegetal matrixes that yield variable recovery rates expressed as mg/g (except when g/L, percentage or m/g expressed as gallic acid equivalents (GAE) or catechin monohydrate equivalents (CME) are indicated), and diverse relative costs (L for low, M for moderate, and H for high) are presented for the extraction of tannins and which may allow their industrial scale up.

Method	Conditions	Source	Recovery (mg/g)	Relative Cost (L, M, H)	Reference
*SLE*	1% NaOH (aq)	*Castanea dentata* peels	4.071	L	[40]
	1% Na_2_SO_3_ (aq)		0.609	L	
	1.5% EtOH (aq)	*Pinus pinaster* barks	62.8 CME	L	[41]
	66% EtOH (aq)	Grape by-products	12.3 g/L	L	[42]
	40% EtOH (aq)	Acorn	80%	L	[52]
	Ionic liquid A	*Acacia catechu*	85%	M/H	[44]
	Ionic liquid B 0.5 M	Grape skin	60.1	M/H	[53]
*SFE*	CO_2_ + EtOH 70%; 40 °C; 10 MPa	*Picea abies* bark	26.38	H	[45]
	CO_2_ + MeOH 40%; 80 °C; 65 MPa	Grape seeds	770	H	[46]
	CO_2_ + EtOH; 50 °C; 18.80 MPa	*Camellia sinensis* leaves	499.90	H	[54]
	CO_2_ + EtOH 10%; 50 °C; 10 MPa	*Pinus pinaster* wood	75.61	H	[55]
	CO_2_, 50 °C, 30 MPa	*Punica granatum* leaves	340	H	[56]
*PWE*	H_2_O; 50 °C; 150 MPa	*Pistacia vera* by-products	70.90	H	[49]
	H_2_O; RT; 250 MPa	*Viola × wittrockiana*	93.86	H	[57]
	H_2_O; 100 °C; 2 MPa	Larch wood	381.90	H	[51]
	H_2_O; 100 °C; 10,34 MPa	Grape pomace	52.90	H	[50]
	H_2_O; 140 °C; 4 MPa	*Lavatera thuringiaca*	72.23 GAE	H	[58]
*ME*	EtOH 70%; 125 W	Grape seeds	528	H	[59]
	MeOH 60%	Grape by-products	22.27 mg/L	H	[60]
	EtOH 45%; 340 W	*Ceratonia siliqua* kibbles	4.11	H	[61]
	H_2_O; 150 W	*Acacia mollissima* barks	47.64	H	[62]
	EtOH; 150 W		30.29		
	EtOH (aq); 400 W	*Galla chinensis*	528.5	H	[43]
	EtOH 96%;	*Lavatera thuringiaca*	71.15 GAE	H	[58]
*UE*	MeOH 90%; 140 W	*Quercus* sp.	127	L	[63]
	EtOH 44%; 500 W	Grape by-products	86.67	L	[64]
	dH_2_O; 301 W	*Phyllanthus amarus*	27.23	L	[65]
	EtOH (aq); 1200 W	*Galla chinensis*	491.20	L	[43]
	EtOH (aq) + ME; 1200 W		543.50		
	Ionic liquid B 2.5 M + ME; 1200 W		630.2		
	EtOH 96%; 216 W	*Lavatera thuringiaca*	71.78 GAE	L	[58]

Room temperature (RT); aqueous dilutions (aq); deionized water (dH_2_O); water (H_2_O); ethanol (EtOH); methanol (MeOH); ionic liquids: A: Dimethylammonium dimethylcarbamate; B: 1-butyl-3-methylimidazolium bromide. Solid/liquid extraction (SLE); superfluid extraction (SFE); pressurized water extraction (PWE); microwave extraction (ME); ultrasonic extraction (UE).
